# Ammonium acid urate urolithiasis in anorexia nervosa: a case report and literature review

**DOI:** 10.1002/ccr3.896

**Published:** 2017-03-31

**Authors:** Mina Fukai, Tetsu Hirosawa, Hideo Nakatani, Tomoko Muramatsu, Mitsuru Kikuchi, Yoshio Minabe

**Affiliations:** ^1^ Department of Psychiatry and NeurobiologyGraduate School of Medical ScienceKanazawa UniversityKanazawaJapan

**Keywords:** Ammonium acid urate urolithiasis, anorexia nervosa, laxative abuse

## Abstract

Ammonium acid urate urolithiasis is a quite rare condition. Our literature review of ammonium acid urate urolithiasis suggests that ammonium acid urate urolithiasis should be regarded as a general medical complication related to anorexia nervosa, and purging by laxative abuse might be a crucially important risk.

## Introduction

Ammonium acid urate (AAU) urolithiasis is a quite rare condition. Its frequency is estimated as 1% among patients with urinary stones 1. However, reports of AAU urolithiasis are increasing in Japan in relation to excessive diets and laxative abuse 2. This report describes the experience of AAU urolithiasis in anorexia nervosa binging–purging subtype (ANBP) and presents discussion of the possible pathological mechanisms related to ammonium acid urate urolithiasis in anorexia nervosa (AN).

## Case Report

The patient, a 39‐year‐old woman, has an 8‐year history of ANBP. Her psychiatric history began at the age of 31, when she engaged in self‐induced vomiting and dietary restriction, leading to weight loss from 50 to 41 kg (167.5 cm; BMI, from 17.8 to 14.5 kg/m^2^). At the age of 37, the patient was admitted to our hospital because of weight loss down to 37.9 kg (BMI 13.5 kg/m^2^). By supervised refeeding, her weight was restored to 45.8 kg (BMI 16.4 kg/m^2^) at discharge.

During regular visits, her weight loss continued after she restarted purging via vomiting and use of laxatives. She also began to restrict her water intake. Over the following 18‐month period, she lost weight to 43.0 kg (BMI, 15.3 mg/m^2^) and presented dehydration. In addition, she repeated mild hypokalemia.

She was referred to our emergency ward because of right back pain. The computed tomography revealed a right ureteral stone (5 mm) and right hydronephrosis. After she was prescribed tiquizium bromide for pain control, she received follow‐up care in the Department of Urology. The stone was radiolucent on the kidney–ureter–bladder X‐ray. After 1 month, she spontaneously passed one stone. The stone was revealed to be an AAU by infrared spectrophotometry.

## Discussion

Urinary stones with AAU have endemic features that are typically observed in economically developing countries 1. The region specificity of this stone formation is related to an acidic purine‐rich and phosphorus‐poor diet combined with low fluid intake and frequent diarrhea 3. However, although AAU urolithiasis is extremely rare in industrialized countries, it is also observed 1.

In Japan, retrospective studies have shown the frequency of AAU calculi in urolithiasis as 0.38% 4. In this study, the authors concluded that, by dividing AAU patients into pure and mixed AAU groups by crystallography, the pure AAU group consisted predominantly of young, thin women who had low levels of serum protein and potassium. These characteristics suggest a relation between AAU urolithiasis and anorexia nervosa (AN). Speaking from an epidemiological perspective 5, the estimated lifetime prevalence of AN is greater among females than among males. Actually, the female/male anorexia incidence rate ratio has been estimated as 8.20. Generally, AN develops in adolescents or young adults. Hypokalemia is presented in those who vomit frequently or who misuse large quantities of laxatives or diuretics 5.

Therefore, we specifically examined clinical cases of AAU urolithiasis in Japan to ascertain the relation between AAU urolithiasis and AN. As expected, of 31 patients with AAU urolithiasis, no fewer than 10 patients were diagnosed as AN 6, 7, 8, 9, 10, 11, 12, 13, 14 where prevalence of AN is 0.6% among the general population 15. Five other patients 6, 16, 17, 18, 19 also exhibited clinical features suggestive of AN, including low body weight, severe dietary restriction, and laxative abuse. A brief summary of these 15 cases is presented in comparison with our case in Table 1.

**Table 1:** Case reports of AAU urolithiasis related to anorexia in Japan

	References	Reported year	Sex	Age	Background	Low body weight	Laxative abuse	Vomiting	Hypokalemia
1	Miyamoto et al. 14	1988	F	28	AN	+	+	−	+
2	Saito et al. 12	1997	F	24	AN	+	UN	UN	+
3	Kato et al. 10	1998	F	26	AN	+	+	−	+
4	Komori et al. 9	2000	F	27	AN	+	UN	+	+
5	Nishio et al. 11	2001	F	20	AN	+	+	+	+
6	Matsuzaki et al. 16	2001	F	25	AN suspected	+	+	−	−
7	Kato et al. 13	2002	F	25	AN	+	+	−	−
8	Nakamura et al. 18	2002	F	32	AN suspected	+	+	−	−
9	Kato et al. 7	2004	F	21	AN	+	+	−	+
10	Kato et al. 7	2004	F	18	AN	+	+	−	+
11	Kato et al. 8	2004	F	27	AN	+	+	−	+
12	Shimizu et al. 19	2006	F	36	AN suspected	+	+	UN	−
13	Shirato et al. 6	2006	F	38	AN suspected	+	+	−	+
14	Shirato et al. 6	2006	F	27	AN	+	−	−	+
15	Watanabe et al. 17	2010	F	34	AN suspected	+	UN	UN	−
16	This case	2016	F	39	AN	+	+	+	+

AAU, ammonium acid urate; AN, anorexia nervosa; UN, unknown; +, have symptoms; −, have no symptoms.

Almost all cases involved laxative abused (Table 1). Dick et al. 20 hypothesized that the development of AAU attributable to the laxatives results from the gastrointestinal loss of water and electrolytes. Loss of potassium and sodium causes intracellular acidosis, which increases ammonia production in urine. Dehydration and loss of sodium induce secretion of aldosterone, which exacerbates hypokalemia and reduces urine volume, resulting in hyperuricemia. Highly concentrated uric acid and ammonia promote AAU stone formation.

Although case 14 (Table 1) 6 involves no laxative abuse, the patient presented remarkably low body weight (BMI 14.1 kg/m^2^), dehydration, and hypokalemia because of restricted intake. This case shows that AN without laxative abuse can develop AAU urolithiasis because of extremely restricted intake of water and potassium.

Vomiting is reportedly associated with hypokalemia 5, but no report of the relevant literature describes a case of AAU urolithiasis caused by vomiting alone (Table 1). Because vomiting induces metabolic alkalosis as a result of loss of acid, the resulting low acid excretion in urine might prevent AAU formation. In fact, past reports have described that patients with AAU calculi caused by laxative abuse or anorexia nervosa showed acidic urine 7.

Results show that AN is associated with numerous general medical complications that are directly attributable to weight loss and malnutrition. Results obtained in this case and those of earlier reports suggest that AAU urolithiasis should be regarded as a general medical complication in AN. Although any subtype of AN can develop AAU urolithiasis, purging by laxative abuse might be a crucially important risk for AAU urolithiasis.

## Authorship

MF: collected data and wrote the first draft of the manuscript. TH, TM, HN, and MK: revised the manuscript critically for important intellectual content.

## Conflict of Interest

The authors declare that they have no conflict of interest related to this report.
